# Evaluation of the quality of information on gouty arthritis on Chinese internet platforms: A cross-sectional comparative analysis

**DOI:** 10.1097/MD.0000000000048496

**Published:** 2026-05-01

**Authors:** Yingqi Gao, Jiabo Zhu, Haitao Wang, Chun Yang, Shuohang Zhang, Jiaqi Guo, Jiaming Liang, Liyan Zhang

**Affiliations:** a Orthopaedic Center, The Affiliated Hospital of Beihua University, Jilin City, Jilin Province, China; b Department of Orthopedics, The Affiliated People’s Hospital of Ningbo University, Ningbo, Zhejiang, China; c School of Basic Medical Sciences, Beihua University, Jilin City, Jilin Province, China; d School of Basic Medical Sciences, Harbin Medical University, Harbin City, Heilongjiang Province, China.

**Keywords:** bilibili, gouty arthritis, quality analysis, short video, TikTok

## Abstract

Acute gout attacks cause severe pain, and short-video platforms have become patients’ primary source of information. However, the quality and reliability of this information are increasingly concerning. This study will systematically evaluate the information quality of gouty arthritis-related content on Bilibili and TikTok video-sharing platforms, along with factors influencing video quality. This study systematically evaluated the quality and reliability of 100 popular gout-related videos each from Bilibili and TikTok. Video quality and reliability were assessed using the global quality score, Modified DISCERN (mDISCERN), JAMA Benchmark Standard, and Hexagonal Radar Schema (HRS) tools. Correlations between video quality and metrics such as likes, comments, saves, and shares were also analyzed. Results showed median scores across 4 metrics on Bilibili: global quality score 3.0 (2.00, 4.00), mDISCERN3..0 (3.00, 4.00), JAMA 3.0 (2.00, 3.00), HRS 5.0 (4.00, 6.00); TikTok’s corresponding scores were 3.0 (IQR 3.00–4.00), 3.0 (IQR 3.00–4.00), 3.0 (IQR 3.00–3.75), and 3.0 (IQR 2.00–4.50). Although Bilibili’s HRS scores were higher than TikTok’s, video quality was generally poor across both platforms. Furthermore, the study found a positive correlation between video length and quality. Increased likes and shares may not always reflect improved video quality, as these metrics can be influenced by the entertainment nature of online videos and may not fully indicate quality. Our research indicates that the health information short videos related to gouty arthritis on Bilibili and TikTok have poor quality, but the videos uploaded by medical professionals are considered reliable in terms of comprehensiveness and content quality. Health information seekers must carefully evaluate the scientific accuracy and reliability of short videos providing medical information on Bilibili and TikTok before making healthcare decisions.

## 1. Introduction

Gout is a common type of crystalline arthropathy which occurs due to deposition of monosodium urate crystals in the joint or in its surrounding tissues. Such deposits will lead to an effusion, pain and swelling in the joints resulting in gouty arthritis (GA) seen in hyperuricemia.^[1]^ Increased serum uric acid levels can cause aggravated inflammation and ultimately formation of tophi, exacerbating the disease.^[2]^ When the joints show recurrence of attacks along with the persistence of swelling and pain, the condition is called chronic GA.^[3,4]^ Due to improvements in the general living conditions, the incidence of GA is increasing year after year with changes in the dietary and habitual practices. Though generally not dangerous by itself, the condition affects so much the quality of the work and standard of living that it imposes a considerable burden on the medical services.

The structure of digital health communication is changing how people obtain health information.^[5,6]^ Digital short-form video platforms with high information density rates, emotional impact and explodable distribution rates are now central to health communication. These advantages divert information dissemination from text formats to video scenario formats.^[7]^ Meanwhile, the enormous numbers of creators with little oversight of the platforms generates concern about the reliability of medical information being disseminated.^[8,9]^

In China, Bilibili and TikTok dominate the short-video industry, with a combined daily active user population of more than 800 million.^[10,11]^ Among the general public and nonprofessionals, gout is often regarded as a “disease related to diet,” and is commonly attributed to overeating, excessive drinking, or indulgence in delicious foods.^[12,13]^ Some media outlets have reinforced this causal relationship through humorous, exaggerated or fictionalized depictions, and labeled gout as a “self-inflicted” disease.^[14,15]^ This may cause patients to feel ashamed, thereby delaying their visits to the doctor.^[16]^ This paper is dedicated to GA for a variety of reasons. The algorithms of social media often create “information silos,” where unverified remedies, especially from laymen, are amplified.^[17,18]^ Further, commercial factors create situations wherein medications are overhyped and dietary supplements marketed in secret.^[19,20]^ The immediate symptomatic relief elicited from attacks of GA produce a notion that cessa-tion of pain is tantamount to cure.^[21]^ Preliminary investigation further reveals that popular videos seldom, if ever, deal with the management of uric acid level. This creates the potential for shortsighted behavior on the part of patients.

To systematically evaluate the multi-dimensional characteristics of video quality, this study employed 4 scoring scales for videos related to GA: The global quality score scale (GQS) and the modified DISCERN Scale (mDISCERN) were used to assess the quality and reliability of the video content. The Journal of the American Medical Association (JAMA) guidelines are used for source authority. The Hexagonal Radar Schema (HRS) provides a simple assessment across 6 criteria such as definitions, symptoms, risk factors, and other parameters. The validity of these tools has been established in prior research.^[22–25]^ This systematic approach explains the balance between scientific accuracy and public engagement, supporting future intelligent content filtering systems.

## 2. Methods

### 2.1. Moral background

This research did not involve the collection of any clinical case data, the use of human biological samples or the handling of laboratory animals. All data analyzed were obtained from public video data from the platforms Bilibili and TikTok. At no point were users identified or private information extracted during the course of the investigation so as to pose a threat of invasion of privacy. The retrospectively based analysis applied only to public existing videos and did not involve interference with user behavior so that there was no necessity for ethical review.

### 2.2. Search strategy and data collection

**Figure 1.**
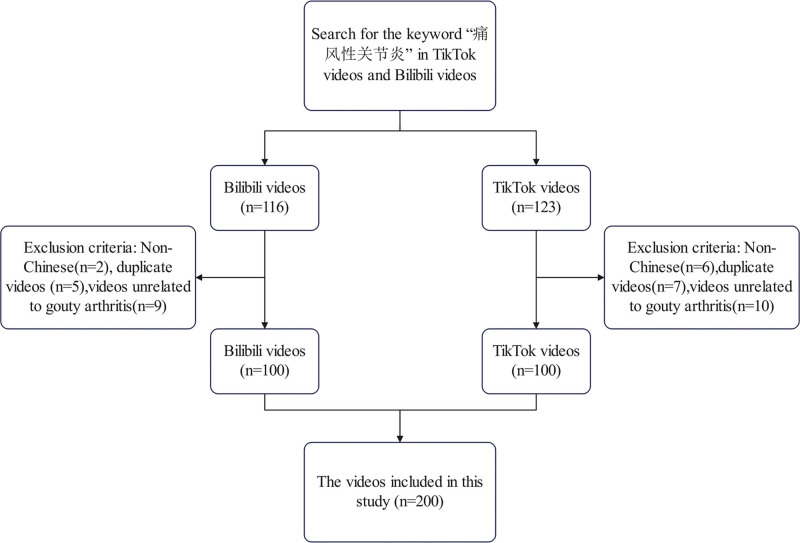
Search strategy for GA short videos. GA = gouty arthritis.

This research had a cross-sectional design. On September 27th, 2025, an ordered form of data collection was performed, using “GA” as the search keyword in the corresponding platforms TikTok (Chinese version 34.0.0) and Bilibili (Chinese version 8.43.0) (see Fig. 1). In order to avoid any disruption caused by personalized recommendation algorithms, the search was made through newly registered blank accounts, where no content filtering had been implemented. Based on previous studies,^[26–29]^ in order to balance the representativeness of the content and the feasibility of the evaluation process, the videos ranked in the top100 on each platform by default were finally selected as the research samples. Inclusion criteria: Chinese videos and videos containing content related to GA. Exclusion criteria: Exclude non-Chinese content; remove videos that are duplicated across multiple platforms; remove videos with irrelevant themes.

Sample size was cut at the 100 most popular videos for each platform. This limit was chosen on the basis of the previous literature which suggested that exceeding this point did not add greatly to the robustness of the conclusions.^[30–32]^ Systematically extract the following video metadata and dissemination metrics: basic attributes: Video title, publisher type (professional physician/nonprofessional physician/other healthcare personnel/science communicator/general user); dissemination effectiveness: Engagement metrics (likes, saves, comments, shares), days since publication, and video duration (seconds). All collected data are archived in structured spreadsheets and managed through standardized data processing using Microsoft Excel.

### 2.3. Video categories

We categorized videos into 2 groups based on the publisher’s certification category: medical professionals and nonmedical professionals. Medical professionals were further subdivided into: professional physician, physician, and other healthcare professionals; nonmedical professionals were further subdivided into: science communicators and general users. The detailed classification information can be found in Table 1.

**Table 1:** Classification of videos.

Publisher’s certification category	
1. Medical professionals	Individuals who have real name recognition and professional accreditation in medical area, including doctors, nurses and other healthcare professionals.
2. Nonmedical professionals	Institutions who do not have professional accreditation in medical area, including science communicators and general users
Medical professionals	
1. Professional physician	Including orthopedic and rheumatology specialists
2. Nonprofessional physician	Including specialists in other surgery, internal medicine, emergency medicine, imaging, interventional medicine and other areas of medicine
3. Other healthcare professionals	Including nurses, epidemiologists, technicians, basic research specialists, and others.
Nonmedical professionals	
1. Science communicators	Institutions who do not have professional accreditation in medical area, including newspaper, TV station, network media and other none-professional groups.
2. General users	Including ordinary users, patients, and their families

### 2.4. Video evaluation

We employed the GQS, Modified DISCERN, JAMA benchmark criteria, and the HRS tool to assess the reliability and information quality of videos, respectively.

Originally developed by Bernard et al for website assessment, the global quality scale (GQS) now also rates video information on a 1 (very low) to 5 (very high) scale.^[23,33]^

Derived from the DISCERN instrument by Singh et al, the modified DISCERN measure allows for the assessment of the reliability of video clips with reference to 5 parameters. The Modified DISCERN tool is more convenient and accurate for the purpose of evaluating video-based materials than the original instrument.^[34,35]^ Each of the 5 items is given a score of 1 to 5 with a higher score corresponding to a better score for trustworthiness.

The JAMA Benchmark judges online source credibility using 4 criteria: authorship, attribution, disclosure, and currency. Each criterion met earns 1 point.

The HRS coding scheme includes 6 domains (definition, signs, risk factors, examination, management, outcomes) which provides an overall framework for health content quality. Each domain is rated from 0 (not existing) to 2 (fully covered). Radar charts illustrate visually the comparative overall performance of the various dimensions of each HRS.

Although these tools were mainly developed for written materials, they are usually applicable to video research as well. Detailed information about the GQS, Modified DISCERN, JAMA and HRS can be found in Supplementary Materials (Tables S1–4, Supplemental Digital Content, https://links.lww.com/MD/R785).

Video links were randomly sorted and submitted to 2 orthopedic physicians for independent assessment. Prior to scoring, evaluators received standardized training covering detailed scoring criteria from GQS, Modified DISCERN, JAMA, and HRS. Each physician independently reviewed the videos on separate devices to complete scoring and classification (by publisher type). If a score discrepancy exceeded 2 points for the same video, a third senior attending physician participated in a review to reach a final consensus through discussion. All scoring data was recorded in Excel spreadsheets, double-entered, and cross-checked to ensure accuracy.

### 2.5. Statistical analysis

The Shapiro–Wilk test was used to assess data normality. Non-normally distributed data were described using the median (interquartile range, IQR), while categorical variables were expressed as frequencies (percentages). Comparisons between 2 groups employed the Wilcoxon signed-rank test, with pairwise comparisons between groups adjusted using Dunn test. Inter-rater reliability was measured using Cohen’s kappa coefficient (*κ*), with *κ* ≥0.8 indicating high agreement.^[36]^ The statistical significance threshold for *P*-values was set at <.05. Spearman rank correlation analysis was employed to investigate correlations among non-normally distributed variables. All statistical analyses were performed using GraphPad Prism version 9.0.0.

## 3. Result

### 3.1. Video characteristics

Based on keyword searches, we obtained 200 videos for data extraction and analysis: 100 from TikTok and 100 from Bilibili. Table 2 shows that TikTok videos received significantly higher numbers of likes, comments, shares, and saves compared to Bilibili videos (all *P* <.001), indicating more active user engagement with TikTok content. Conversely, videos on Bilibili were significantly longer in duration than those on TikTok. The difference was statistically significant (*P* <.001).

**Table 2:** Video characteristics related to GA on Bilibili and TikTok.

Characteristic	Bilibili (n = 100), median (IQR)	TikTok (n = 100), median (IQR)	*P*
Number of likes	17.0 (3.25, 110.25)	1363.5 (227.00, 10377.50)	<.001
Number of comments	3.0 (0.00, 24.50)	84.0 (12.25, 459.00)	<.001
Number of collections	12.5 (2.00, 78.00)	451.5 (63.25, 3756.75)	<.001
Number of shares	8.0 (1.25, 43.25)	419.0 (47.00, 3390.50)	<.001
Time since upload (d)	511.0 (327.25, 928.25)	194.0 (35.25, 535.00)	<.001
Video duration (s)	182.0 (105.00, 337.50)	73.5 (53.25, 101.50)	<.001
GQS score	3.0 (2.00, 4.00)	3.0 (3.00, 4.00)	.271
mDiscern score	3.0 (3.00, 4.00)	3.0 (3.00, 4.00)	.416
JAMA score	3.0 (2.00, 3.00)	3.0 (3.00, 3.75)	<.001
HRS score	5.0 (4.00, 6.00)	3.0 (2.00, 4.50)	<.001

GA = gouty arthritis, GQS = global quality score, HRS = hexagonal radar schema, IQR = interquartile range, JAMA = Journal of American Medical, mDISCERN = modified DISCERN.

**Figure 2.**
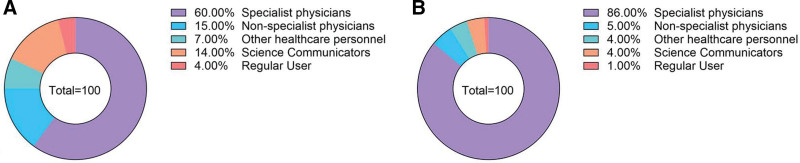
Percentage distribution of GA videos by different publishers on Bilibili and TikTok. (A) Publisher distribution for Bilibili videos. (B) Publisher distribution for TikTok videos. GA = gouty arthritis.fi

As shown in Table 3, on Bilibili, professional doctors uploaded the highest number of videos (60/100, 60%), followed by nonprofessional doctors (15/100, 15%), science communicators (14/100, 14%), other healthcare personnel (7/100, 7%), and general users (4/100, 4%). As shown in Table 4, medical videos on TikTok are highly concentrated among professional doctors (86/100, 86%), followed by nonprofessional doctors (5/100, 5%), science communicators (4/100, 4%), other healthcare professionals (4/100, 4%), and ordinary users (1/100, 1%), who represent an extremely low proportion. The specific distribution situation is shown in Figure 2.

**Table 3:** Video characteristics of different sources on Bilibili.

Variable	Specialist physicians (n = 60)	Non specialist physicians (n = 15)	Other healthcare personnel (n = 7)	Science communicators (n = 14)	Regular user (n = 4)
Video sources (n = 100), median (IQR)					
Likes	13.0 (3.00, 76.50)	10.0 (1.00, 127.00)	147.0 (7.00, 450.00)	35.5 (5.50, 2417.50)	4.5 (0.75, 220.50)
Comments	2.5 (0.00, 16.75)	1.0 (0.00, 20.00)	25.0 (1.00, 47.00)	2.0 (1.00, 344.50)	0.0 (0.00, 68.25)
Collections	9.0 (2, 45.75.00)	8.0 (2.00, 79.00)	363.0 (1.00, 746.00)	59.0 (17.50, 1080.00)	1 (0.00, 119.75)
Shares	7.0 (1.00, 18.25)	11.0 (1.00, 20.00)	151.0 (8.00, 520.00)	31.0 (7.75, 923.00)	1.5 (1.00, 35.00)
Time since upload (d)	438.0 (251.75, 894.75)	642.0 (342.00, 1120.00)	375.0 (264.00, 664.00)	854.0 (388.75, 1161.25)	618.0 (351.75, 851.25)
Video duration (s)	174.5 (98.25, 262.50)	105.0 (87.00, 206.00)	463.0 (131.00, 1370.00)	618.0 (248.50, 975.00)	211.0 (70.75, 817.75)
GQS score	3.0 (3.00, 3.00)	2.0 (2.00, 3.00)	2.0 (2.00, 3.00)	4.0 (3.00, 4.25)	3.0 (3.00, 3.00)
mDISCERN score	3.0 (3.00, 4.00)	3.0 (2.00, 3.00)	3.0 (2.00, 3.00)	3.5 (2.75, 4.25)	3.5 (3.00, 4.00)
JAMA score	3.0 (2.00, 3.00)	2.0 (2.00, 3.00)	2.0 (2.00, 3.00)	3.0 (2.00, 3.00)	3.0 (3.00, 3.00)
HRS score	5.0 (4.00, 5.50)	4.5 (3.00, 6.00)	5.0 (5.00, 6.00)	6.0 (5.38, 8.00)	6.5 (3.00, 8.50)

GQS = global quality score, HRS = hexagonal radar schema, IQR = interquartile range, JAMA = Journal of American Medical, mDISCERN = modified DISCERN.

**Table 4:** Video characteristics of different sources on TikTok.

Variable	Specialist physicians (n = 86)	Non specialist physicians (n = 5)	Other healthcare personnel (n = 4)	Science communicators (n = 4)	Regular user (n = 1)
Video sources (n = 100), median (IQR)					
Likes	1626.5 (281.00, 7490.00)	1890.0 (112.00, 16250.00)	149.0 (78.75, 377.50)	77000.0 (9077.50, 189000.00)	18.0 (18.00, 18.00)
Comments	91.5 (11.75, 453.00)	170.0 (13.50, 402.50)	15.0 (12.50, 34.75)	4112.0 (405.75, 8265.25)	3.0 (3.00, 3.00)
Collections	459.0 (70.75, 2082.00)	328.0 (37.00, 5198.50)	39.5 (14.00, 83.75)	21500.0 (3602.50, 34500.00)	7.0 (7.00, 7.00)
Shares	472.5 (59.75, 3323.00)	134.0 (56.50, 3842.00)	26.5 (10.25, 136.50)	48000.0 (5841.25, 80750.00)	4.0 (4.00, 4.00)
Time since upload (d)	163.0 (31.75, 463.00)	135.0 (50.00, 780.00)	562.0 (142.75, 691.75)	466.5 (252.50, 1702.00)	1265.0 (1265.00, 1265.00)
Video duration (s)	73.5 (54.75, 101.00)	64.0 (44.50, 95.00)	68.0 (39.50, 105.50)	127.0 (55.00, 587.50)	45.0 (45.00, 45.00)
GQS score	3.0 (3.00, 4.00)	3.0 (2.50, 3.50)	3.0 (3.00, 3.00)	3.0 (3.00, 4.50)	2.0 (2.00, 2.00)
mDISCERN score	3.0 (3.00, 4.00)	3.0 (3.00, 3.00)	3.5 (3.00, 4.00)	3.0 (2.25, 4.50)	3.0 (3.00, 3.00)
JAMA score	3.0 (3.00, 4.00)	3.0 (3.00, 3.00)	3.0 (3.00, 3.75)	3.5 (3.00, 4.00)	3.0 (3.00, 3.00)
HRS score	3.0 (2.00, 4.63)	2.5 (2.00, 5.25)	3.25 (2.25, 3.88)	3.75 (3.125, 7.00)	2.5 (2.50, 2.50)

GQS = global quality score, HRS = hexagonal radar schema, IQR = interquartile range, JAMA = Journal of American Medical, mDISCERN = modified DISCERN.

### 3.2. Online video quality analysis

**Figure 3.**
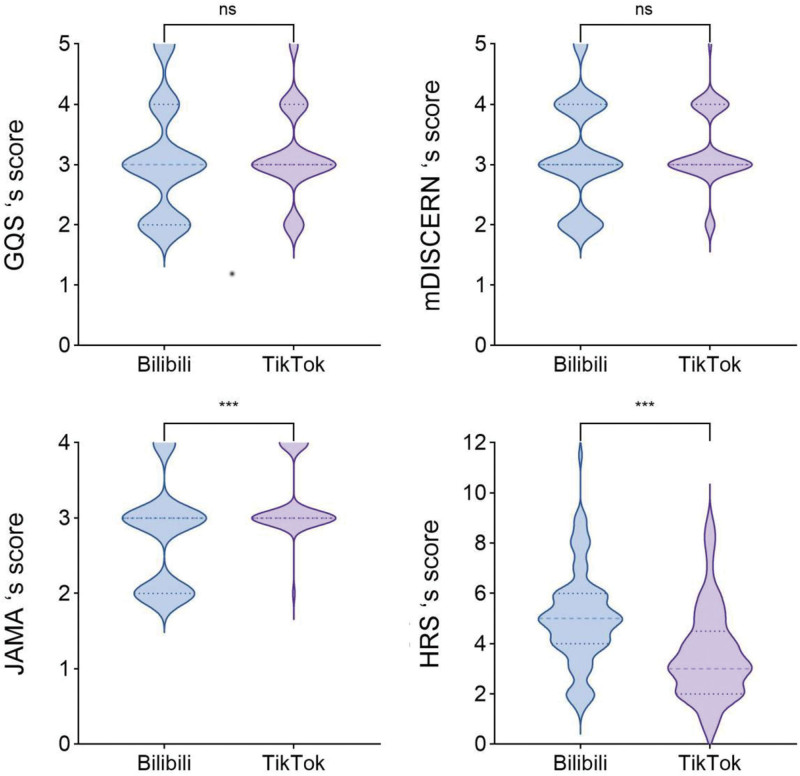
Scores for GQS, mDISCERN, JAMA, and HRS of short videos related to GA on Bilibili and TikTok. (A) Comparison of GQS scores between Bilibili and TikTok videos. (B) Comparison of mDISCERN scores for Bilibili and TikTok videos. (C) Comparison of JAMA scores for Bilibili and TikTok videos. (D) Comparison of HRS scores for Bilibili and TikTok videos. GQS = global quality score, HRS = hexagonal radar schema, JAMA = Journal of American Medical, mDISCERN = modified DISCERN.

The *κ* values for the 2 observers in this study were 0.81, indicating high inter-observer reliability. The study shows that for videos on the Bilibili platform, the median GQS score is 3.0 (IQR 2.00–4.00), the median mDISCERN score is 3.0 (IQR 3.00–4.00), the median JAMA score is 3.0 (IQR 2.00–3.00), and the median HRS score is 5.0 (IQR 4.00–6.00); while for videos on the TikTok platform, the median scores of GQS, mDISCERN, JAMA and HRS are 3.0 (IQR 3.00–4.00), 3.0 (IQR 3.00–4.00), 3.0 (IQR 3.00–3.75) and 3.0 (IQR 2.00–4.50), respectively. Although Bilibili’s HRS was higher than TikTok’s, the overall video quality and reliability on both platforms were moderately rated. Table 2 and Figure 3 indicate no significant differences in GQS and Modified DISCERN scores between platforms (*P* = .271 and *P* = .416, respectively), whereas significant differences exist for JAMA and HRS scores (*P* <.001).

**Figure 4.**
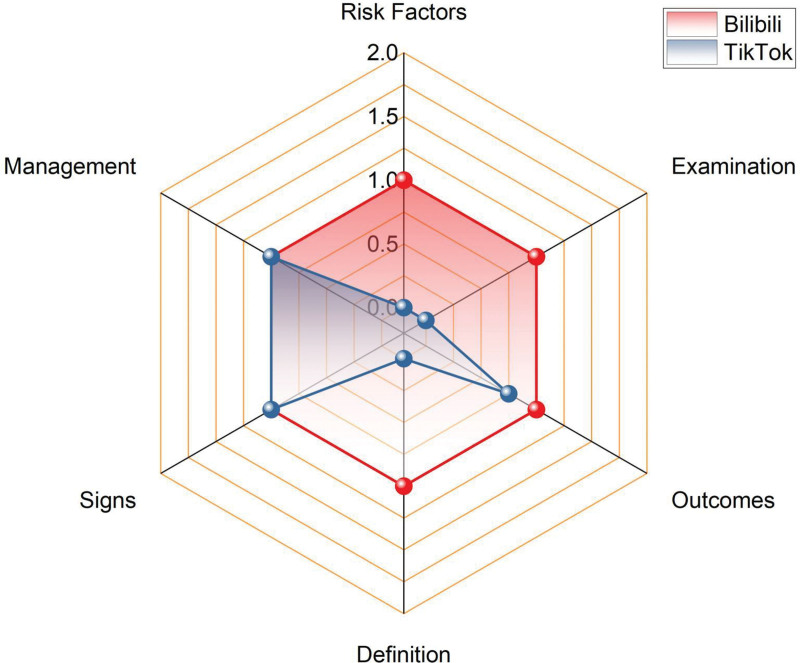
The HRS scores for each part of Bilibili and TikTok. HRS = hexagonal radar schema.

Figure 4 indicates that across the 6 dimensions of HRS scores, Bilibili outperformed TikTok in screening, diagnosis, risk factors, and disease outcomes, scored equally on symptoms and management, and had the lowest diagnosis scores across all platforms.

### 3.3. Correlation analysis

**Figure 5.**
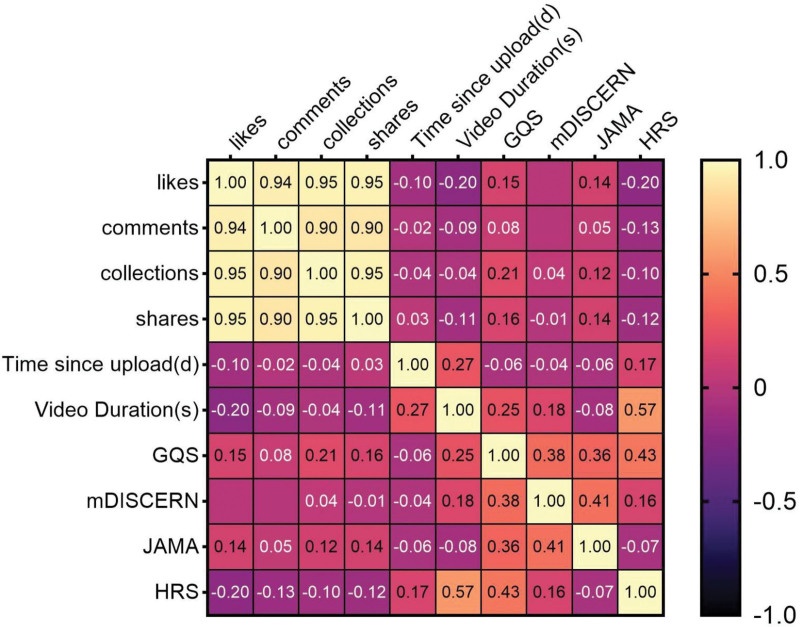
Heatmap of variable correlation matrices showing Spearman correlation analysis between different video variables, GQS, mDISCERN, JAMA, and HRS scores. Blank cells indicate correlations < 0.01. GQS = global quality score, HRS = hexagonal radar schema, JAMA = Journal of American Medical, mDISCERN = modified DISCERN.

The data is not normally distributed; therefore, we employed Spearman correlation analysis to reveal relationships among different video variables. Figure 5 shows: there exists a significant strong positive correlation among dissemination effects, reflecting the synergistic nature of user interaction behaviors (e.g., high likes readily drive comments, saves, and shares, forming a closed-loop of interactive dissemination). The upload date and temporal sequence of videos had no significant impact on interactions. Additionally, apart from duration, interaction variables such as likes and comments showed no significant linear correlation with quality scores.

Video duration is a significant factor influencing GQS and Modified DISCERN scores, with longer videos receiving higher-quality ratings. Video duration (in seconds) shows a significant positive correlation with HRS, indicating that longer videos perform better across all 6 dimensions of health information.

## 4. Discussion

### 4.1. Key findings

This cross-sectional study examined the characteristics of gout-related video content on the 2 most used Chinese short-video platforms, Bilibili and TikTok. Overall, video quality was poor across both platforms. Low quality likely resulted from little in the way of uploading restrictions and low moderating of content. Although no significant difference in video quality was observed between the 2 platforms (GQS, *P* = .271; mDISCERN, *P* = .416), TikTok video quality appears to be slightly lower than Bilibili’s. This may be related to the fact that videos on Bilibili tend to be longer, allowing for more comprehensive dissemination of disease-related information. However, longer videos alone do not guarantee higher-quality ratings.^[26]^ The professionalism of video sources has a significant impact on the quality of content and the effectiveness of its dissemination. While TikTok relies on professional doctors to generate high engagement, its video sources are relatively limited. Bilibili, on the other hand, has a more diverse range of video sources, enabling it to produce richer and more varied content. Therefore, viewers need to be critical of the scientific reliability of gout-related videos when utilizing the platforms for obtaining health information.

### 4.2. Correlation between video quality and video features

This study reveals the complex nonlinear relationship between video quality and dissemination effectiveness on short-video platforms. Consistent with earlier studieswe did not find any salient relationship between these 2 aspects.^[32,37,38]^ Indicators such as likes and comments have limited utility in assessing medical accuracy. More notably, metrics for dissemination effectiveness may exacerbate the complexity of misinformation propagation, underscoring the necessity for algorithmic optimization.^[39]^ Algorithmic bias in this respect is particularly marked in health: completely educational videos without any multimodal attributes (such as music and visual tricks) do not stimulate sufficient audience attention, so the distinction between high and low quality is blurred.^[40,41]^

### 4.3. Evaluation of quantitative scoring tools

The coherence of mDISCERN and GQS when rating the quality of the information provided by videos is found to be satisfactory. However, it should be noted that mDISCERN and GQS are primarily designed to judge the information quantity provided by the videos and are not able properly to judge the intrinsic quality of the video material. In addition, it was found that the JAMA benchmark, which contains but 4 questions, is probably unable to give proper guidance regarding the reliability of the knowledge provided, corroborating previous reports.^[42]^ Similarly, the HRS has been illustrated in a radar chart form that provides a more intuitive comparison of the information provided on the 2 platforms.

### 4.4. Limitations

This study has several limitations. Firstly, this study exclusively collected Chinese video data from the TikTok and Bilibili platforms. All samples were cross-sectional and obtained at a single point in time, with a limited sample size. Given the dynamic nature of the platform’s recommendation algorithm and its sensitivity to account history, cross-sectional data captured at a single point in time (September 27, 2025) struggles to capture the temporal evolution of content visibility. Future research should adopt a multi-time-point, multi-account sampling design to further validate the robustness of the findings.^[43]^ Secondly, the 4 evaluative tools used in this study were designed specifically for textual media and may not be the best fit for assessing audiovisual content. For example, while the number of comments was analyzed, the content of the comments was not examined in depth. Sometimes, viewers leave comments not to express praise or agreement, but to voice dissatisfaction. These negative comments can also contribute to an increase in the overall number of comments. Future research should assess comment types (e.g., positive, negative, requests for help) to determine if this correlates with video quality, in particular, whether answers to viewers’ inquiries are useful.

## Author contributions

**Data curation:** Yingqi Gao, Jiabo Zhu, Shuohang Zhang, Jiaming Liang.

**Funding acquisition:** LIyan Zhang, Haitao Wang.

**Methodology:** Yingqi Gao, Jiabo Zhu.

**Supervision:** LIyan Zhang.

**Writing – original draft:** Yingqi Gao, Jiabo Zhu, Chun Yang, Shuohang Zhang, Jiaqi Guo, Jiaming Liang.

**Writing – review & editing:** LIyan Zhang, Haitao Wang.

## Supplementary Material
