# Supplementary material for: Evaluation of the quality of information on gouty arthritis on Chinese internet platforms: A cross-sectional comparative analysis
Source: Medicine (Baltimore). 2026 May 1;105(18):e48496. doi: 10.1097/MD.0000000000048496 (PMC13138388; doi:10.1097/MD.0000000000048496)
Supplement: Supplementary file 1 [file medi-105-e48496-s001.pdf]

Supplementary Table 1. Description of the Global Quality Score scale.

Scale	Description
Poor quality (1 point)	Poor quality and poor flow of the site, most information missing, not at all useful for patients
Generally poor quality (2 point)	Generally poor quality and poor flow, some information listed but many important topics missing, of very limited use to patients
Moderate quality (3 point)	Moderate quality, sub-optimal flow, some important information is adequately discussed but others poorly discussed, somewhat useful for patients
Good quality (4 point)	Good quality and generally good flow, most of the relevant information is listed, but some topics not covered, useful for patients
Excellent quality (5 point)	Excellent quality and excellent flow, very useful for patients

Supplementary Table 2. Description of the modified DISCERN score.

Criteria<br>(1 point for each)	Description
Unreliable (1 point)	Is the video clear, concise, and understandable?
Less reliable (2 point)	Are valid sources cited?
Generally reliable (3 point)	Is the content presented balanced and unbiased?
Reliable (4 point)	Are additional sources of content listed for patient reference?
Very reliable (5 point)	Are areas of uncertainty mentioned?

Supplementary Table 3. The *Journal of American Medical Association* benchmark criteria.

Criteria (1 point for each)	Description
Authorship	Authors and contributors, their affiliations, and relevant credentials should be provided
Attribution	References and sources for all content should be listed clearly, and all relevant copyright information should be noted
Currency	Dates when content was posted and updated should be indicated
Disclosure	Conflicts of interest, funding, sponsorship, advertising, support, and video ownership should be fully disclosed

Supplementary Table 4. Description of the Hexagonal Radar Schema

Category	Criteria	Score
Definition	Defining a disease or a particular stage, type, category of the disease	Not addressed at all = 0<br>Slightly addressed = 0.5<br>Partially addressed = 1<br>Quite well addressed = 1.5<br>Fully addressed = 2
Signs	Typical signs caused by the disease	
Risk factors	Factors that might cause the incidence of the disease or accelerate its progress	
Examination	Means used for diagnosing and evaluating the disease	
Management	Treatment	
Outcomes	Prognosis of the disease, complications, survival or death	
